# Crosstalk tolerance analysis of coupled-line structures using least square-support vector machine technique

**DOI:** 10.1038/s41598-023-42728-4

**Published:** 2023-09-16

**Authors:** Mohammad G. H. Alijani, Mohammad H. Neshati

**Affiliations:** Electrical Department, Ferdwosi University of Mashhad, Mashhad, Iran

**Keywords:** Engineering, Electrical and electronic engineering

## Abstract

In this paper, crosstalk sensitivity analysis of a microwave coupled-line structure due to the fabrication imperfections is investigated using Least Square-Support Vector Machine (LS-SVM) method. Since LS-SVM uses a set of linear equations instead of a convex quadratic programming problem, the computational cost is extremely reduced compared to that of the well-known Monte Carlo (MC) analysis or even Support Vector Machine (SVM) without decreasing the accuracy. Using this method, the geometrical parameters of the coupled-line are assumed to be randomly distributed using the Latin Hypercube function and the variation range of each parameter is set to ± 50% around its central value. The frequency response of the coupled-line is estimated and compared with those of the measured and simulation ones for a few well-known practical case studies. The results show that the LS-SVM procedure quickly predicts the worst-case crosstalk expectation values and accurately anticipates the probability of obtaining various outcomes of the coupled-line for the specified parameter variation over a wide frequency range.

## Introduction

It is well-known that the most important features of microwave and millimeter wave devices and circuits are their sensitivity to the physical and geometrical parameters of the structure. This is due to fabrication imperfections in the manufacturing process and other uncontrollable parameters leading to uncertain device characteristics^1^. Moreover, these phenomena initiate significant variations in the required response and in turn, it is needed to accurately predict the sensitivity analysis of the structure along the design procedure. For electromagnetic applications, sensitivity analysis, design exploration, and optimization are essential during the design process^2,3^.

The tolerance analysis is conventionally done using Monte Carlo (MC) method or its improved variants, which can be undoubtedly regarded as the standard reference technique for this purpose^4^. The next-generation of electrical and electronic equipment will consist of several complex units. The calculating process includes hundreds or in some cases thousand times of the MC procedure to consider tolerance analysis, which requires a long time. Although the MC process provides high accuracy, it demands a high computational cost without providing a parametric surrogate of the system response. Nowadays, several parametric and statistical techniques, including Parameterized Macro-Modeling (PM)^5^, Polynomial Chaos (PC)^6^, Support Vector Machine (SVM), Generative Models (GM)^7^, and deep learning method^8–10^, have been introduced to investigate the sensitivity analysis of generic microwave components.

Since Least Square-Support Vector Machine (LS-SVM) allows building compact parametric surrogates of nonlinear system responses with several uncertain parameters, it has gained extensive attention over the past years^11^. The LS-SVM is established based on the kernel-based learning techniques, while SVM is a robust methodology for solving problems providing nonlinear rules. In LS-SVM, the final solution of the problem is obtained by finding a set of linear equations instead of a convex Quadratic Programming (QP) problem for typical SVMs^12^, and this is the most important feature of this method. This led to significantly reducing the running time, while the accuracy is comparable with those of the other methods.

In this paper, a systematic approach based-on machine learning method is presented to study the sensitivity analysis associated with the crosstalk arising between the microstrip coupled lines. A surrogate model is established for the coupled structures using the LS-SVM method. Then, for specified tolerances of the geometrical and physical parameters of the structure under study, the frequency response is determined and compared with those of the measured and simulation ones. The results show that the LS-SVM method predicts the target values with acceptable accuracy.

## Uncertainty quantification using LS-SVM

The least-square approximation of linear functions to a specified data set is called Linear Least Square (LLS) problem. The specified data set may be obtained from experimental or simulation results. The LLS expression refers to a set of formulations, which are used to solve a statistical problem. This problem involved linear regression, including variants for weighted or unweighted and correlated residuals. The LLS problem is solved using the inverting matrix of the characteristic equations and orthogonal decomposition methods. The sum of squared residuals is minimized by LLS method and a closed-form formula is obtained for the estimated value of the unknown vector **x** given by Eq. (1), in which **B** is a vector, which holds the observation data set, and **x** is a square or non-square matrix. The *ij* element of **x** is the *i*th observation data of the *j*th independent variable.

1 $$ {\hat{\mathbf{x}}} = pinv({\mathbf{A}}){\mathbf{B}} $$

In addition, *pinv*(.) shows the Pseudo-inverse or the Moore–Penrose inverse operator. The LLS method finds an exact or approximated solution of the problem by minimizing the error vector **e** given by (2).

2 $$ {\mathbf{e}} = \left\| {{\mathbf{Ax}} - {\mathbf{B}}} \right\|^{2} $$

In Eq. (2), the notation ||.||^2^ represents the norm-2 operator and it is defined by (3).

3 $$ \left\| {{\mathbf{Ax}} - {\mathbf{B}}} \right\|^{2} = \left[ {\left( {{\mathbf{Ax}} - {\mathbf{B}}} \right)^{T} \left( {{\mathbf{Ax}} - {\mathbf{B}}} \right)} \right] $$

The combination of LSM and SVM methods is used to define the minimization problem using Eq. (4)^13^, subject to the equality constraints given by Eq. (5).

4 $$ \min \, \Upsilon \left( {{\mathbf{v}},b,e} \right){ = }\frac{1}{2}\left( {\left\| {\mathbf{v}} \right\|^{2} + \zeta \sum\nolimits_{i = 1}^{N} {e_{i}^{2} } } \right) $$

5 $$ y_{i} \left[ {{\mathbf{v}}^{T} \psi (x_{i} ) + b} \right] = 1 - e_{i} , \, \,\,\,\,\,i = 1, \, ..., \, N $$

It is assumed that the total number of training data sets is *N*. It should be noted that the training data set {(*x*_*i*_, *y*_*i*_)}_*i*=1, …, *N*_ is provided by a generic nonlinear system, which is modeled by *y* = *M*(*x*). Also, the input vector of the system is depicted by **x** = [*x*_*1*_, …, *x*_*d*_] ∈ ℝ^*d*^. The system is modeled using a nonlinear SVM regression given by (6).

6 $$ M_{SVM} ({\mathbf{x}}) = \left\langle {{\mathbf{v}},\Psi ({\mathbf{x}})} \right\rangle + b $$

In system modeling by SVM, a non-linear mapping Φ(**x**) = [*ψ*_*1*_(**x**), …, *ψ*_*D*_(**x**)] is applied, which maps a linear space with the dimension of *d* into another space with dimension *D* as the corresponding feature space given by Φ(**.**): ℝ^*d*^ → ℝ^*D*^. Furthermore, the unknown coefficients of the nonlinear regression are depicted by vector **v** ∈ ℝ^*D*^.

For this problem, the bias function and the inner product in ℝ^*D*^ are designated by *b* ∈ ℝ and < **v**, Ψ(**x**) > respectively. To show the trade-off between the system model accuracy and its flatness, a pragmatic factor *ζ* is used. It should be noted that to measure the accuracy, the error variable is defined by *e*_*i*_ ∈ ℝ^14^.

It can be seen that the mentioned formulation for the LS-SVM classifier implicitly corresponds to a regression interpretation with binary targets *y*_*i*_ =  ± 1 using *y*_*i*_^2^ given by (7).

7 $$ \sum\limits_{i = 1}^{N} {e_{i}^{2} } = \sum\limits_{i = 1}^{N} {\left\{ {y_{i} - \left[ {{\mathbf{v}}^{T} \psi (x_{i} ) + b} \right]} \right\}^{2} } $$

The error parameter *e*_*i*_ would also be acceptable for least-squares data fitting so that the same end outcome holds for the regression process. Thus, the LS-SVM classifier formulation is equivalent to Eqs. (8a) to (8c).

8a $$ \Upsilon \left( {{\mathbf{v}},b,e} \right){ = }E_{w} + \zeta E_{D} $$

8b $$ E_{v} = \frac{1}{2}\left\| {\mathbf{v}} \right\|^{2} $$

8c $$ E_{D} = \frac{1}{2}\sum\nolimits_{i = 1}^{N} {e_{i}^{2} } = \frac{1}{2}\sum\nolimits_{i = 1}^{N} {\left\{ {y_{i} - \left[ {{\mathbf{v}}^{T} \psi (x) + b} \right]} \right\}^{2} } $$

The parameter *ζ* is set to tune the amount of regularization versus the sum squared error. So, the hyper-parameter *ζ* is an important factor in determining the solution of the problem. In other words, by tuning this parameter, the accuracy of the final results is changed. Hence, determining the optimum value of *ζ* is an important parameter for researchers, which can be used to provide a Bayesian interpretation to LS-SVM. The solution of the LS-SVM problem is determined using the Lagrangian function given by (9).

9 $$ \begin{gathered} L_{2} ({\mathbf{v}},b,e,\alpha ) = \Upsilon ({\mathbf{v}},e) - \sum\limits_{i = 1}^{N} {\alpha_{i} \left\{ {\left[ {{\mathbf{v}}^{T} \psi (x_{i} ) + b} \right] + e_{i} - y_{i} } \right\}} = \frac{1}{2}{\mathbf{vv}}^{T} + \frac{\zeta }{2}\sum\limits_{i = 1}^{N} {e_{i}^{2} } - \hfill \\ \sum\limits_{i = 1}^{N} {\alpha_{i} \left\{ {\left[ {{\mathbf{v}}^{T} \psi (x_{i} ) + b} \right] + e_{i} - y_{i} } \right\}} \hfill \\ \end{gathered} $$

In the above equation, *α*_*i*_ ∈ ℝ is Lagrange multipliers and the conditions to obtain the optimum solution are determined by Eqs. (10a) to (10d)^11^.

10a $$ \frac{{\partial L_{2} }}{\partial v} = 0 \, \to {\text{ v}} = \sum\limits_{i = 1}^{N} {\alpha_{i} \psi (x_{i} )} $$

10b $$ \frac{{\partial L_{2} }}{\partial b} = 0 \, \to \, \sum\limits_{i = 1}^{N} {\alpha_{i} } = 0 $$

10c $$ \frac{{\partial L_{2} }}{{\partial e_{i} }} = 0 \, \to \, \alpha_{i} = \zeta e_{i} , \, i = 1, \, ..., \, N $$

10d $$ \frac{{\partial L_{2} }}{{\partial \alpha_{i} }} = 0 \, \to \, y_{i} = {\mathbf{v}}^{T} \psi (x_{i} ) + b + e_{i} , \, i = 1, \, ..., \, N $$

By eliminating parameters **v** and *e,* a linear system of equations is obtained instead of a quadratic programming problem given by (11).

11 $$ \left[ {\begin{array}{*{20}c} 0 & {1_{N}^{T} } \\ {1_{N} } & {\Omega + \zeta^{ - 1} I_{N} } \\ \end{array} } \right]\left[ {\begin{array}{*{20}c} b \\ \alpha \\ \end{array} } \right] = \left[ {\begin{array}{*{20}c} 0 \\ Y \\ \end{array} } \right] $$

In (11), *Y* = [*y*_*1*_, …, *y*_*N*_]^T^, 1_N_ = [1, …, 1]^T^, *α* = [*α*_*1, …,*_* α*_*N*_]^T^ and *I*_*N*_ is an *N* × *N* identity matrix. Also, Ω ∈ ℝ^*N*×*N*^ is the kernel matrix, which is defined by (12).

12 $$ \Omega_{ij} = \psi (x_{i} )^{T} \psi (x_{i} ) = K(x_{i} ,x_{j} ) $$

In the above equation, *K*(*x*_*i*_,*x*_*j*_) shows the kernel operator. The kernel matrix is the gram matrix of the kernel “*K*” obtained by evaluating the kernel function on all the couples of input training samples (similar to the covariance matrix). So far, a few kernel types, including linear, polynomial, and Radial Basis Function (RBF) or Gaussian are used, while each of them provides its own unique characteristics^13^. For these kernels, we have:

1. Linear: *K*(x_i_, x_j_) = x_i_^T^x_j_;
2. Polynomial of order *p*: *K*(x_i_, x_j_) = (1 + x_i_^T^x_j_)^*p*^;
3. Gaussian: *K*(x_i_, x_j_) = exp(-||x_i_-x||^2^);

## Application test case

The microstrip coupled-line structures are widely used in microwave applications, including Lange-Coupler, high-speed data link, etc.^15–17^. Tolerance of different geometrical parameters of the applied lines has an adverse effect on the performance of these structures. For example, in a high-speed data link, fabrication imperfections affect the data throughput. So, tolerance analysis has to be considered in the design procedure. In this section, application of the LS-SVM in sensitivity analysis of a few practical uniforms and non-uniform microwave coupled-lines including microstrip line and stripline on the amount of crosstalk is investigated.

In all the provided examples, the per unit length value of the geometrical parameters are defined by *y*(*x*) = *y*_*0*_[1 + 0.5*x*], in which *y*(*x*) and *y*_*0*_ are the physical value of the structure parameter including the fabrication tolerance and its central value, respectively. In addition, *x* is distributed by the Latin Hypercube function in interval [− 1, 1]. So, this representation confirms a variation range of ± 50% around the central value of the parameters. It should be noted that we used 50% to stress the method performance. But, 50% is a huge variability for the uncertainty quantification. Usually, it models the uncertain parameters as Gaussian variables with a standard deviation of 5% or 10% computed with respect to the corresponding mean values depending on the application. In other words, the worst case is considered in the paper. If the model works correctly for the worst case, it will definitely provide a high accuracy answer for the other cases as well.

Our study shows that the 100 numbers of samples (*N*_*S*_ = 100) is enough for a fast convergence rate of the LS-SVM process with a specified acceptable accuracy. But, to obtain a highly accurate result, a maximum 150 numbers of samples are sufficient. So, the applied models are trained using the upper limit of the sample numbers *N*_*S*_. It should be noted that the number of training samples depends on how many input parameters we are considering, their variability, and the complexity of the parametric function we are trying to model. However, it may need a larger number of training samples for other cases. Usually, the training process is started with a small number of data sets, and if an acceptable answer is not obtained, the number of training data sets is increased.

In addition, the crosstalk Probability Density Function (PDF) in each case is derived and compared with those obtained by MC method. Moreover, the simulation results are provided by High Frequency Structure Simulator (HFSS) and considering S-parameter.

### Case study I

In this case, a symmetrically coupled-stripline of length 160 mm, width of 1 mm, dielectric constant of *ε*_*r*_ = 4.7, the distance between the lines 4 mm and ground plane spacing 3.2 mm is considered, while four ports of the coupled-lines are terminated by 50 Ω loads. Also, the per unit length capacitances and inductances are equal to *C*_*c0*_ = *C*_*v0*_ = 125.02 pF/m, *C*_*m*_ = 2.5 pF/m, *L*_*c0*_ = *L*_*v0*_ = 0.4 μH/m, *L*_*m*_ = 8.35 nH/m.

**Figure 1:**
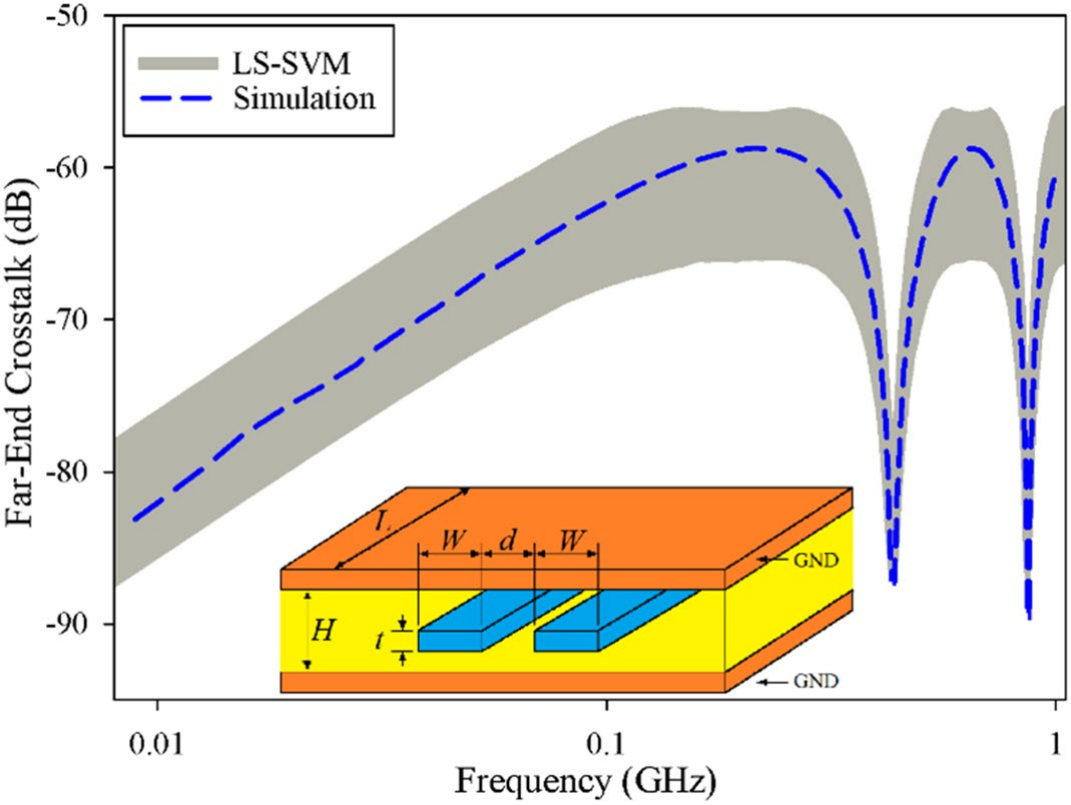
The predicted and simulated far-end crosstalk of a symmetrical coupled-stripline, case I, uncertain area (gray area) using the LS-SVM method, simulated results (dashed line) by HFSS.

**Figure 2:**
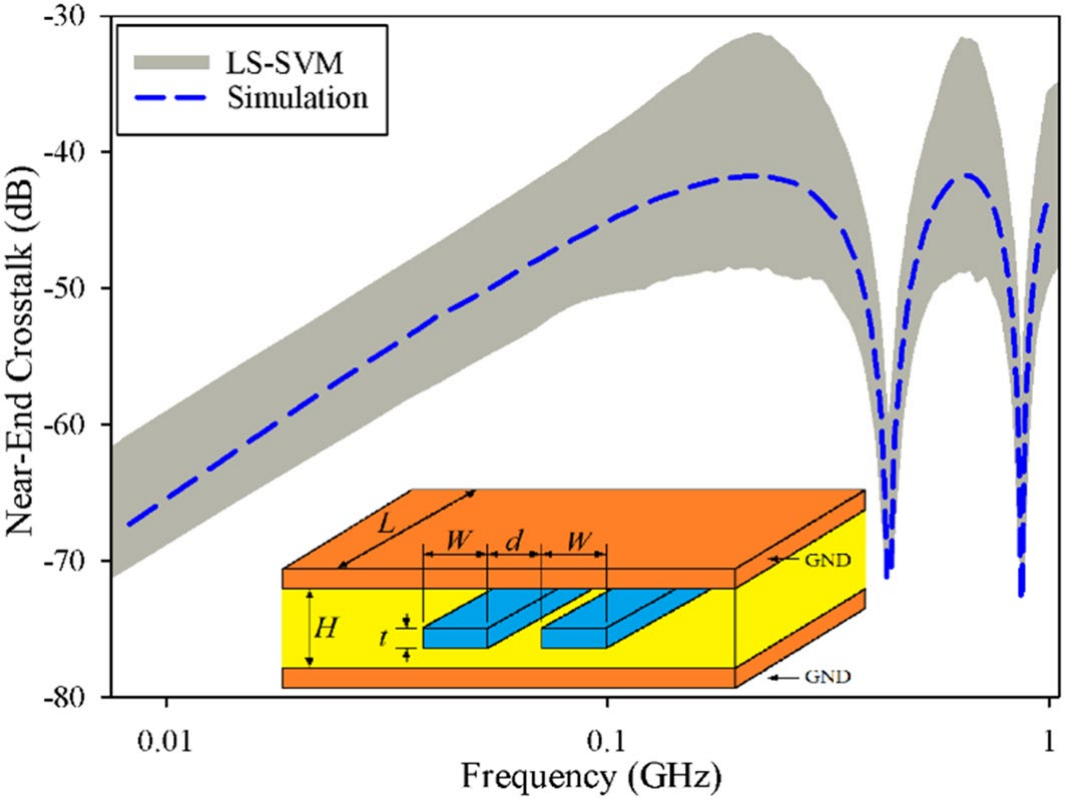
The predicted and measured near-end crosstalk of a symmetrically coupled-stripline, case I, uncertain area (gray area) using the LS-SVM method, simulated results (dashed line) by HFSS.

The obtained results for this case, including the far-end and near-end crosstalk obtained by the LS-SVM (a gray area) and simulation results using HFSS (dashed blue line) are plotted in Figs. 1 and 2. It can be seen that the far-end crosstalk is smoother than that of the near-end one over the considering frequency ranges, whereas the coupling is stronger. These figures show that the LS-SVM method can predict the maximum range variation of the crosstalk with an acceptable accuracy over the specified frequency range.

### Case study II

**Figure 3:**
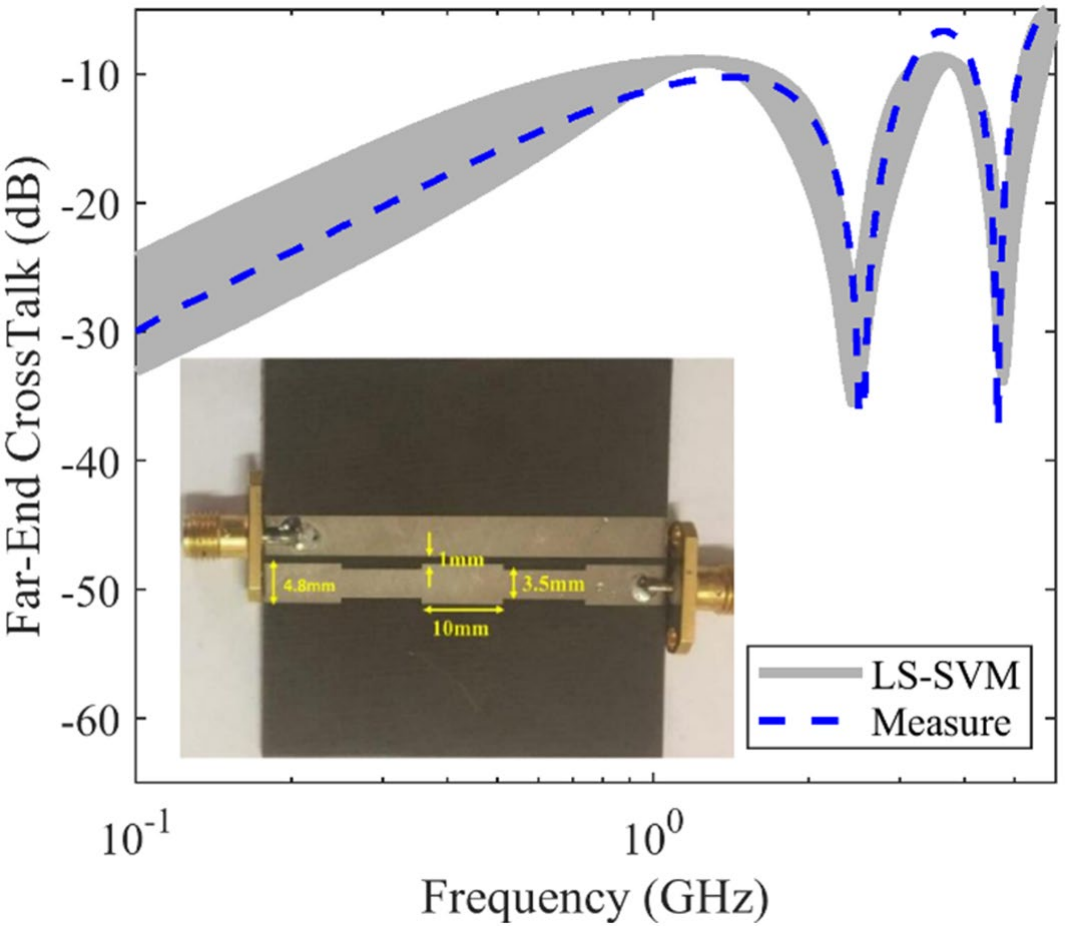
The predicted and measured far-end crosstalk of an asymmetrical non-uniform microstrip coupled-line, case II, uncertain area (gray area) using the LS-SVM method, measured results (dashed line).

In the second example, an asymmetrical non-uniform microstrip coupled line is considered. The applied substrate is Taconic TLY30 with dielectric constant of ε_*r*_ = 2.2 and thickness of *h* = 1.56 mm. The other geometrical parameters are shown in Fig. 3. To achieve maximum coupling in this structure, the two output ports are terminated by open circuit. A machine-based model for this non-uniform coupled-line is established, and the obtained results of crosstalk are plotted in Fig. 2 including the measured results over the frequency range from 0 to 6 GHz. It can be seen that for this example, the maximum range variation of crosstalk is lower than that of the uniform structure.

### Case study III

**Figure 4:**
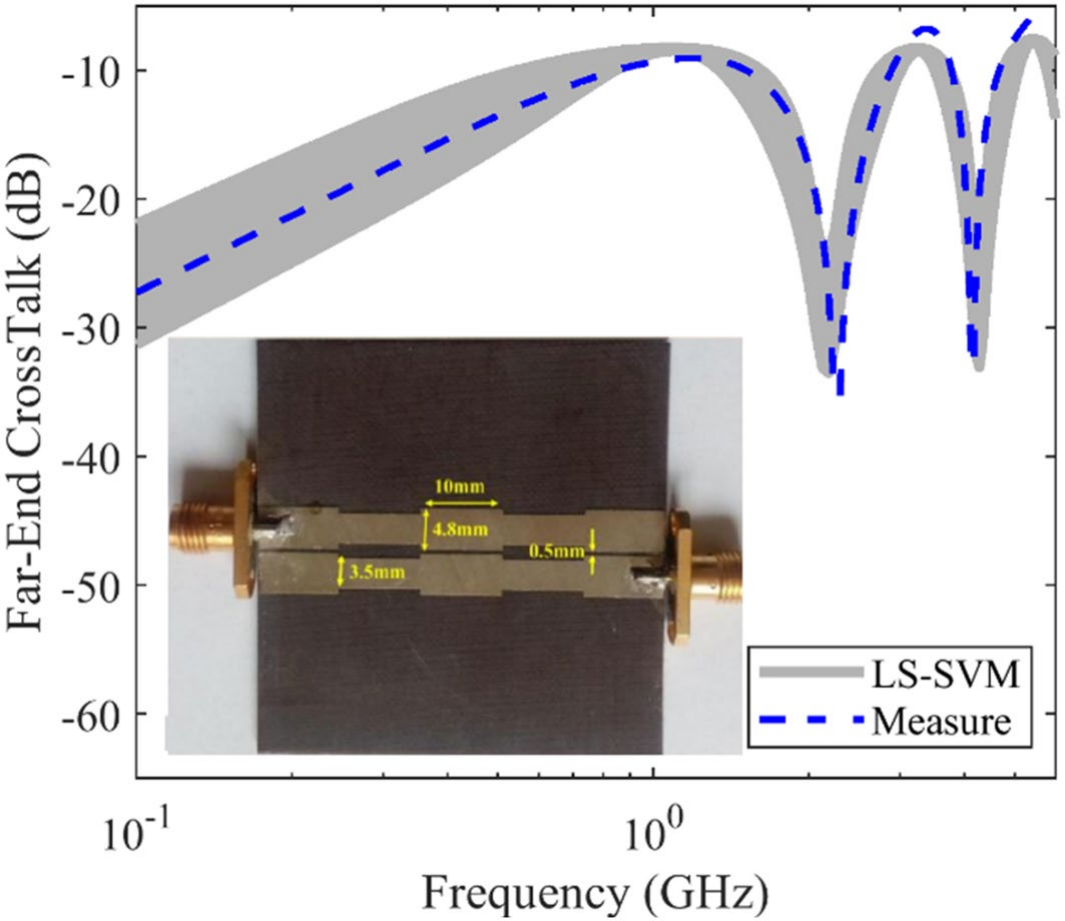
The predicted and measured far-end crosstalk of a symmetrical non-uniform microstrip coupled-line, case III, uncertain area (gray area) using the LS-SVM method, measured results (dashed line).

A symmetrical non-uniform microstrip coupled-line is considered as the third example. Similar to the previous one, the substrate is made of Taconic TLY30 substrate with *ε*_*r*_ = 2.2 and *h* = 1.56 mm, and the other geometrical parameters are shown in Fig. 4. The response of this structure is obtained after establishing the required machine-based model. The obtained results of the crosstalk are plotted in Fig. 4 versus frequency from 0 to 6 GHz, including the measured ones. It can be seen that the maximum range variation of the crosstalk for this structure is similar to that of the asymmetrical microstrip coupled-line.

### Case study IV

**Figure 5:**
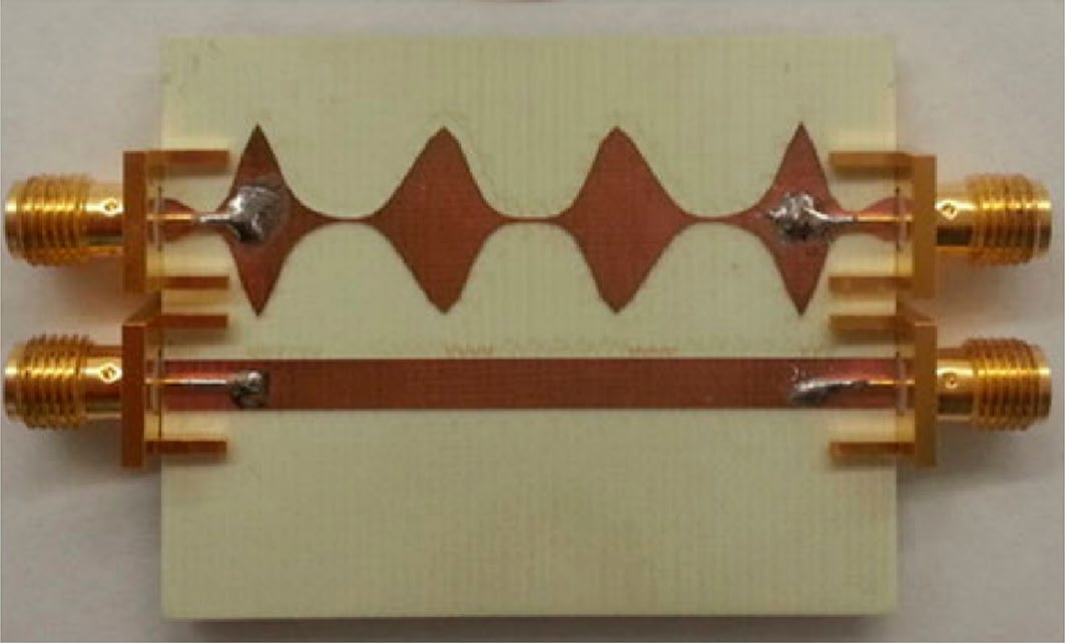
The fabricated configuration of the non-uniform coupled lines^18^.

**Figure 6:**
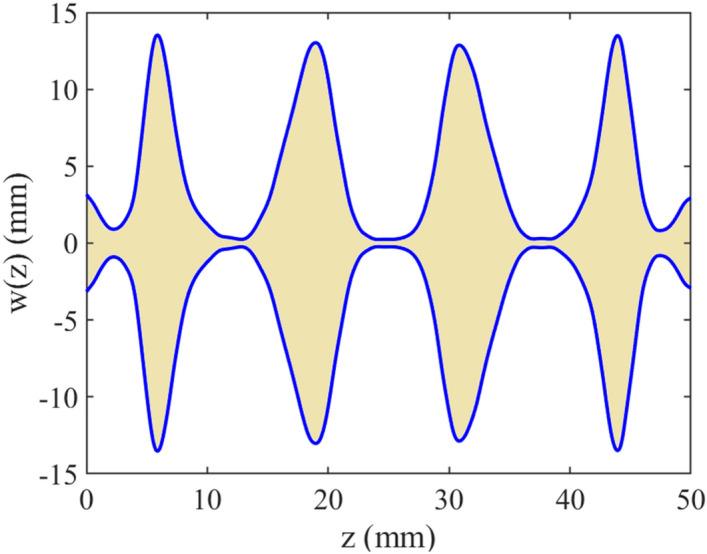
The profile of the non-uniform line of the final case.

In the final case, a microstrip coupled-line with non-uniform profile is considered. The substrate and conductor of this structure are made of RO4835 with relative permittivity of 3.66 and copper with conductivity of 5.8 × 10^7^ S/m, respectively. The length of the two coupled traces and the substrate thickness are about 50 mm and 1.524 mm, respectively. Two coupled lines are terminated to 50 Ω load. The fabricated configuration and the profile of the non-uniform line are depicted in Figs. 5 and 6, respectively^18^.

**Figure 7:**
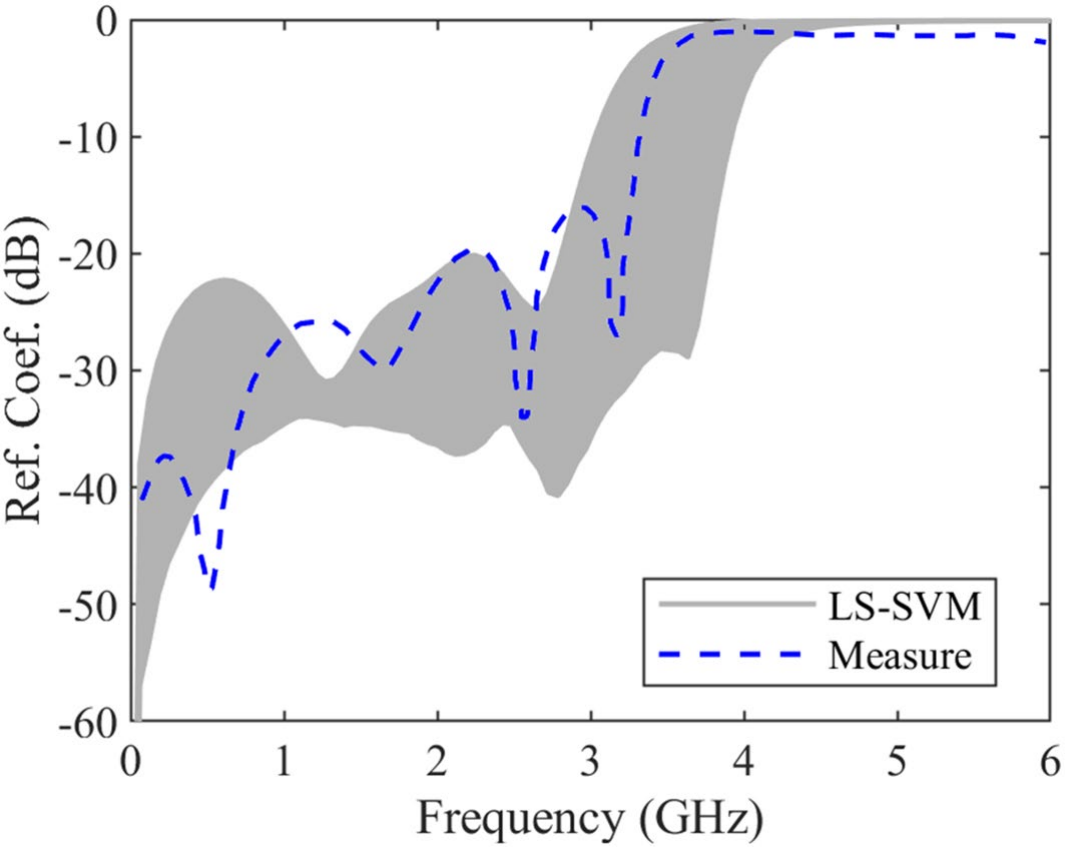
The predicted and measured reflection coefficient of a microstrip coupled-line, case IV, uncertain area (gray area) using the LS-SVM method, measured results (dashed line)^18^.

**Figure 8:**
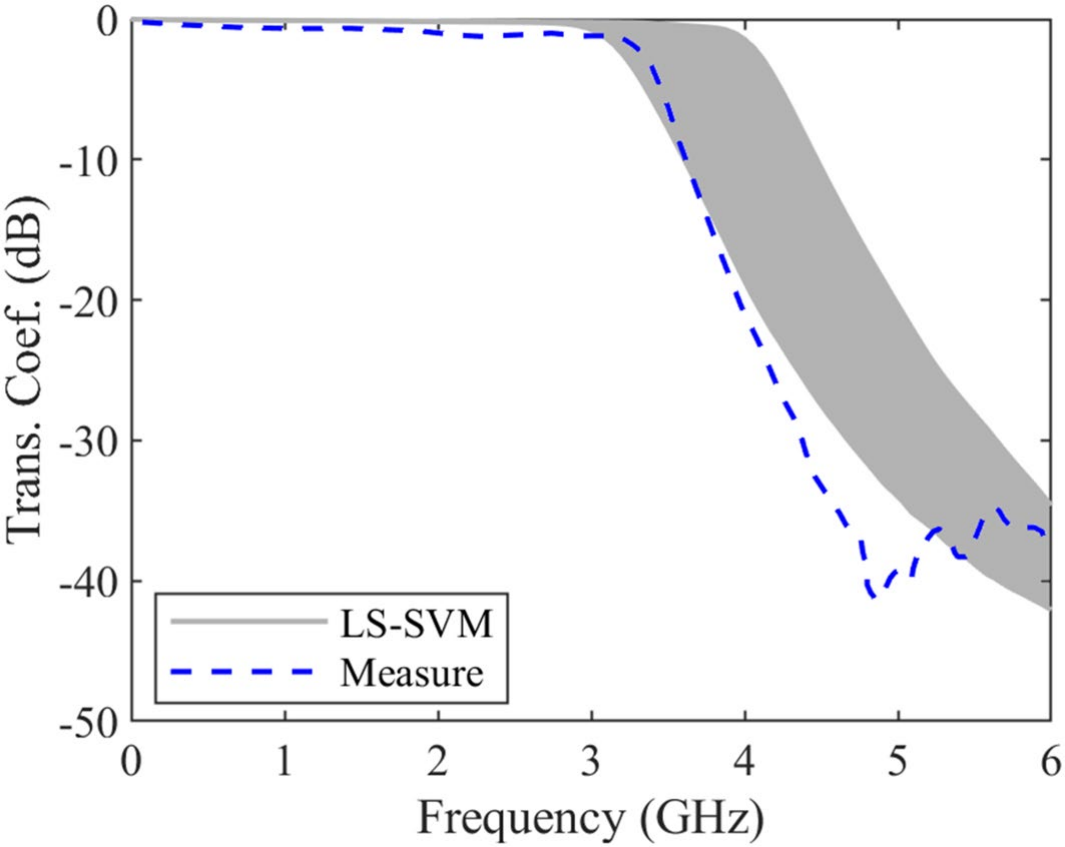
The predicted and measured transmission coefficient of a microstrip coupled-line, case IV, uncertain area (gray area) using the LS-SVM method, measured results (dashed line)^18^.

It should be noted that the data sets for training procedure are produced using the introduced method in^19^. The obtained results of the under-studying coupled line including reflection coefficient and transmission coefficient uncertain area (a gray area) and measured ones (dashed blue line)^18^ are plotted in Figs. 7 and 8 versus frequency, respectively. These results show that the worst-case reflection coefficient and transmission coefficient due to manufacturing imperfection in designing procedures, while the structure parameters change uncontrollably. It means that any changes in the geometrical and physical parameters of the structure in the worst-case lead to these results. The discrimination between the results is because of two reasons. First, a small deviation is produced due to the fabrication error. Using the approximated formulas to generate the training data set is the second reason.

It should be noted that in the present examples, there are several data sets. If there were only two data sets, the global difference measure (GDM) in the feature selective validation (FSV) could be useful to determine the correlation level between the measurement results and the results obtained by the LS-SVM^20^.

**Figure 9:**
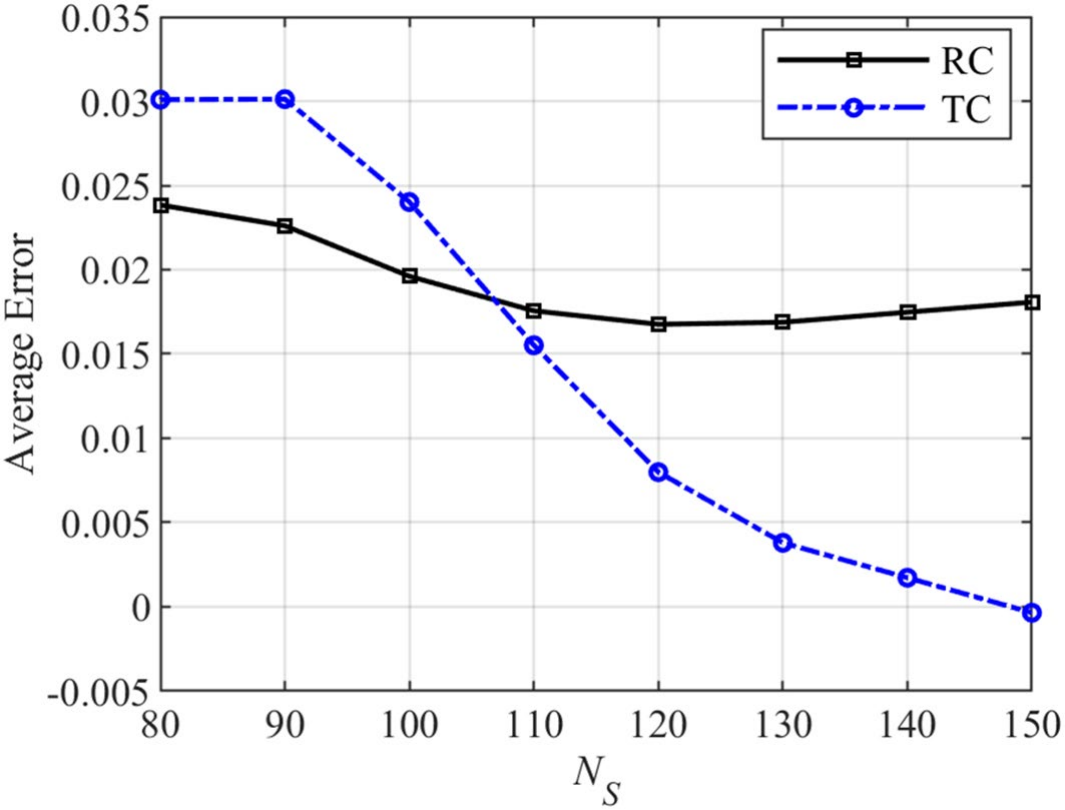
The average error with respect to the number of training data samples (*N*_*S*_) of the last example.

It is clear that the accuracy of the surrogate model depends on the number of the training data samples *N*_*S*_. This issue is shown in Fig. 9. In this figure, the average error of the established surrogate model based-on the LS-SVM method is plotted with respect to the number of the training data samples for the last example. As expected, the average error decreases when the number of the training data samples increases. For all examples, the number of the training data samples can be optimized using the design-of-experiment (DoE) method. More details on this issue can be found in^20^. This issue can be further studied in future work.

### Crosstalk PDF test case

**Figure 10:**
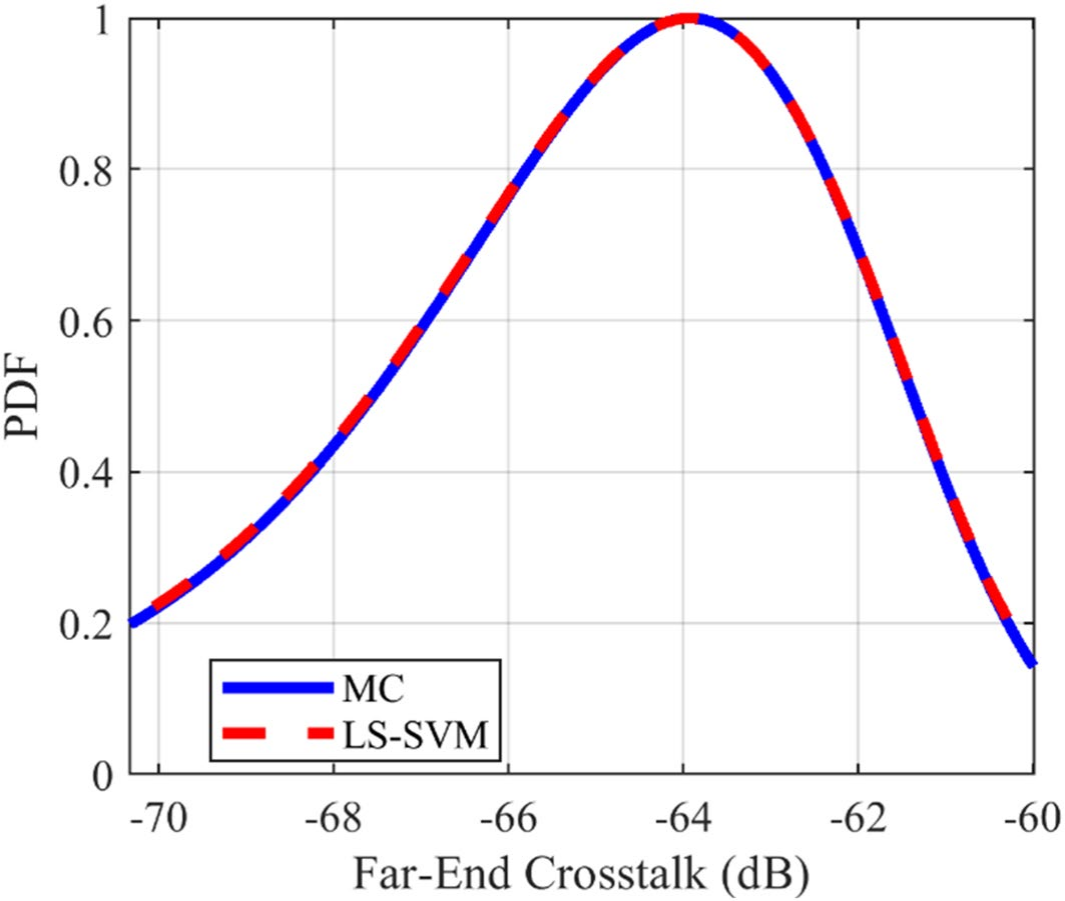
The evaluated far-end crosstalk PDF for the uniform coupled-stripline, case I.

**Figure 11:**
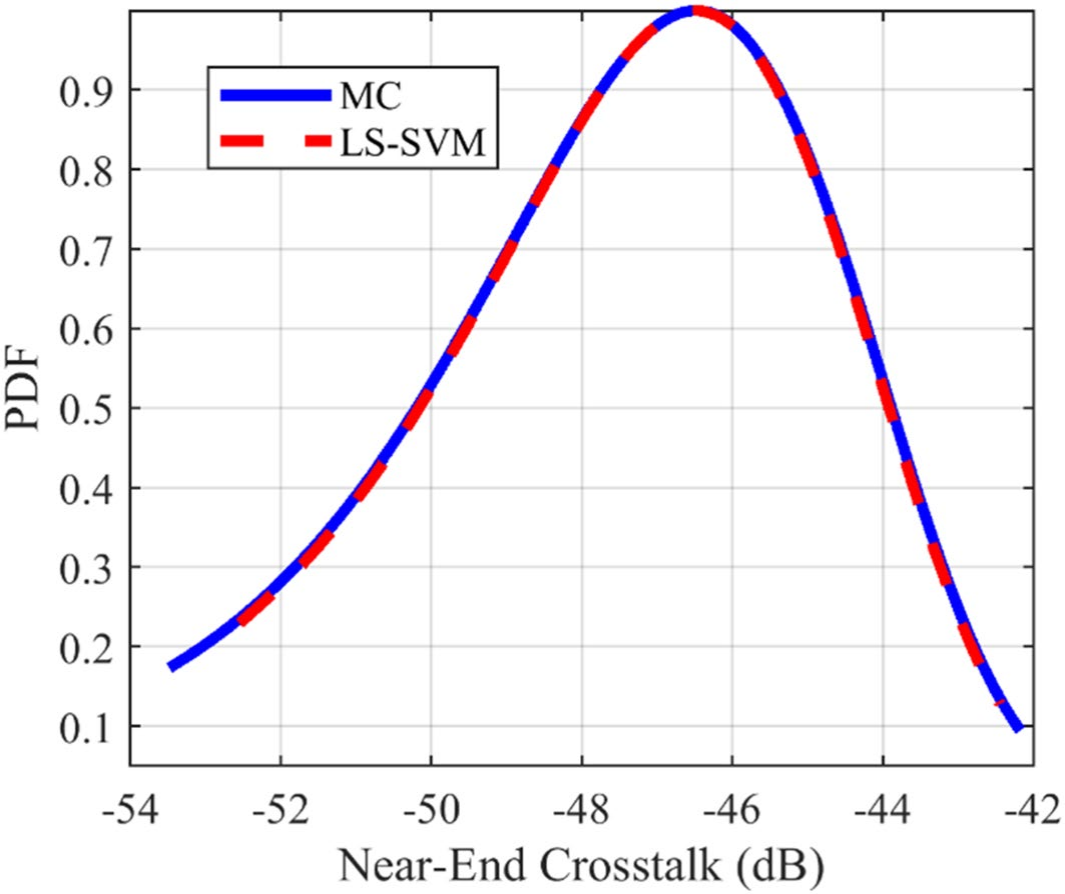
The evaluated near-end crosstalk PDF for the uniform coupled-stripline, case I.

**Figure 12:**
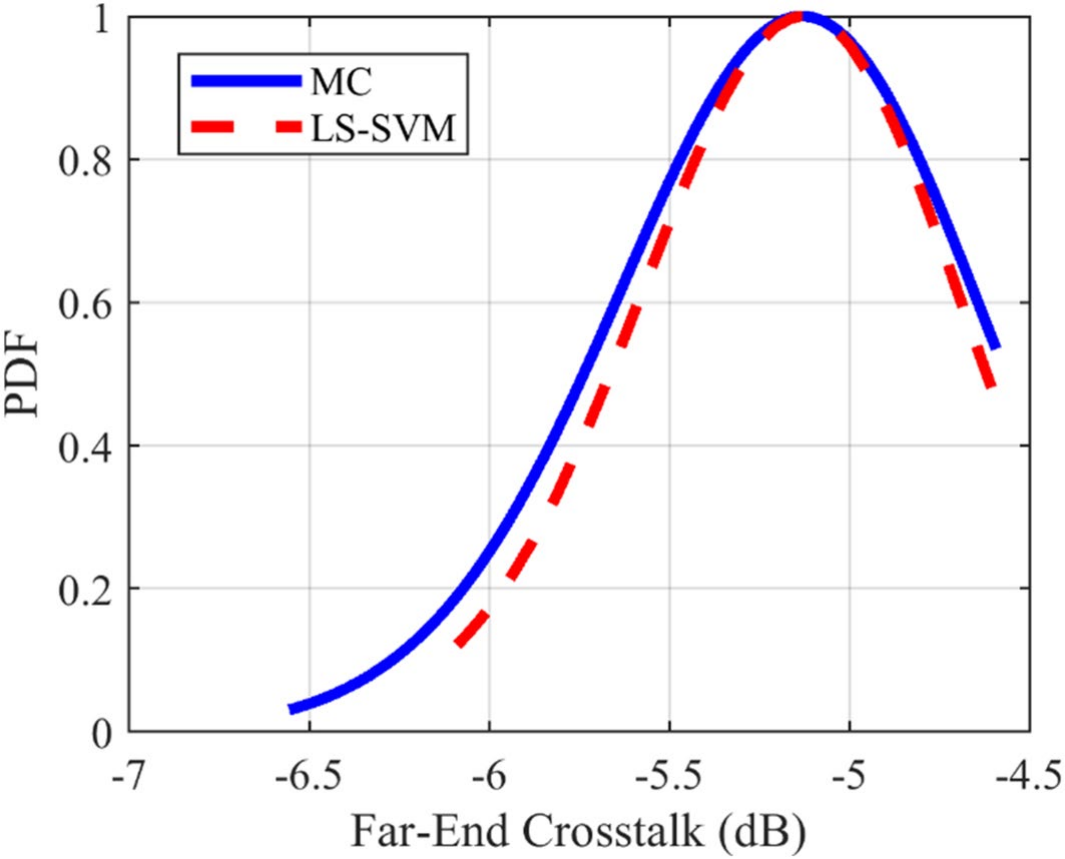
The evaluated far-end crosstalk PDF for the asymmetrical non-uniform microstrip line, case II.

**Figure 13:**
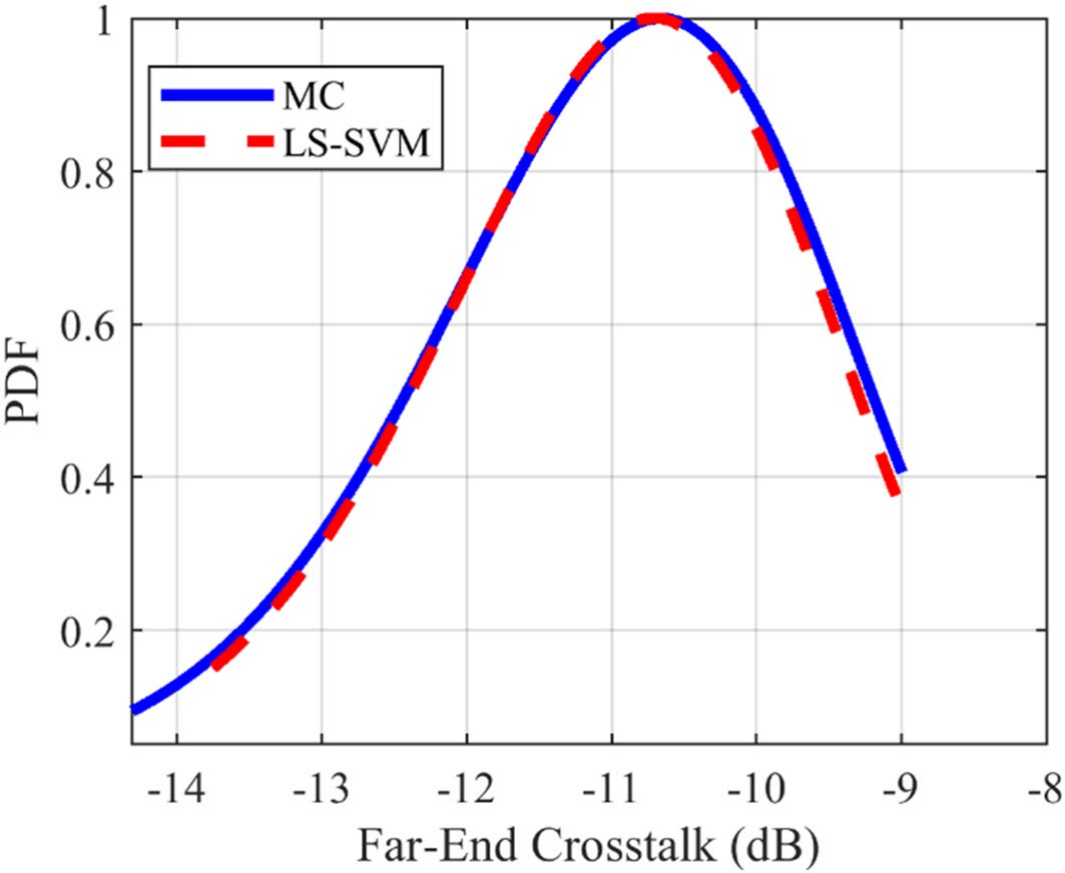
The evaluated far-end crosstalk PDF for the symmetrical non-uniform microstrip line, case III.

**Figure 14:**
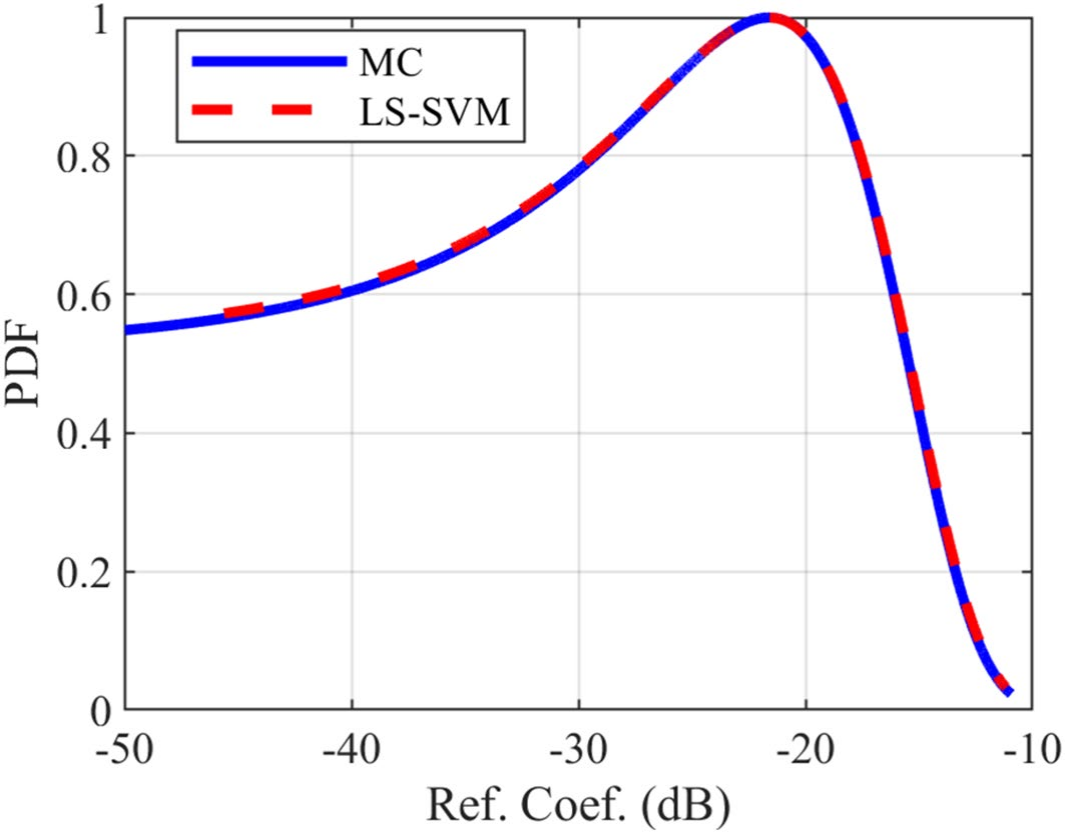
The evaluated reflection coefficient PDF for the non-uniform microstrip line, case IV.

**Figure 15:**
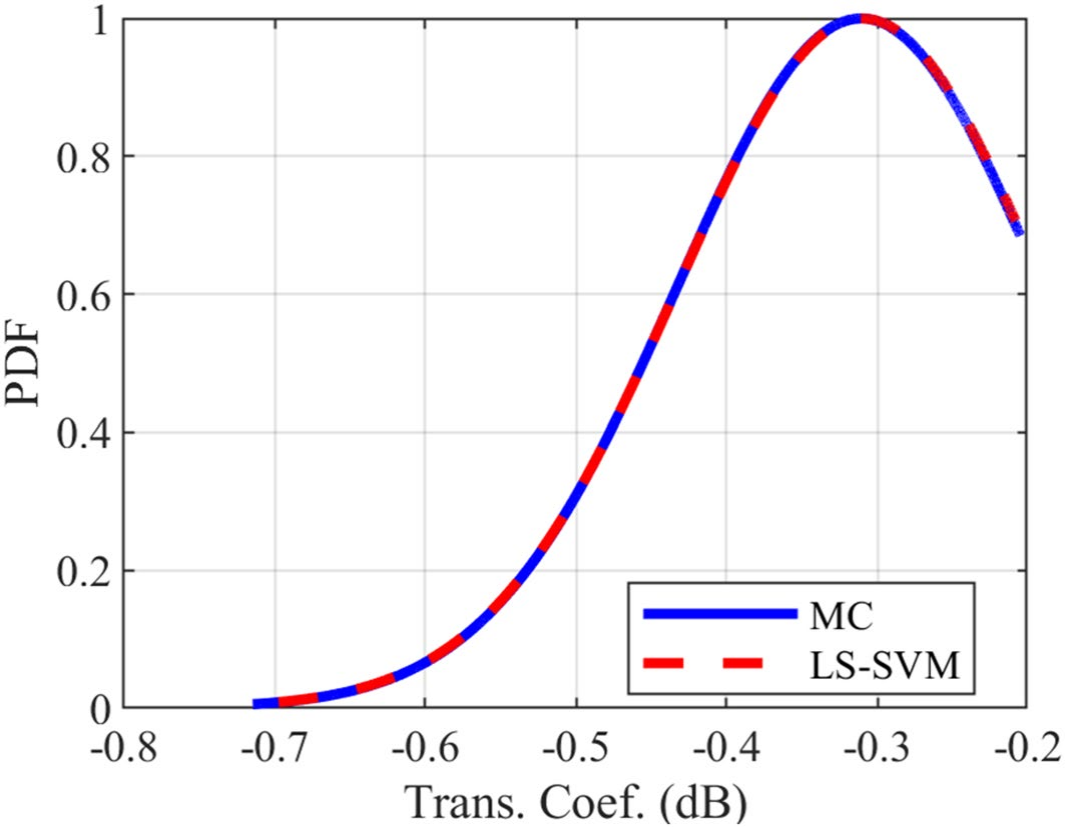
The evaluated transmission coefficient PDF for the non-uniform microstrip line, case IV.

The provided surrogate models are employed to compute the Probability Density Function (PDF) of the crosstalk for the under-studying structures. For all of the mentioned structures, the LS-SVM method with the RBF kernel is used. The comparison of the calculated PDFs of the investigated structures using LS-SVM and Monte Carlo (MC) methods are plotted in Figs. 10, 11, 12, 13, 14, 15 for different structures. Moreover, the PDF of the output crosstalk for all cases is obtained using 30,000 MC iterations. It is clear that the computational cost for the LS-SVM method is significantly lower than that of the applied MC method with specified iterations; however, the accuracy of the LS-SVM method to predict the related PDF is very close to that of the MC model.

Since the last case is a complicated structure, and in order to check the efficiency of the proposed method, this case is examined using different machine learning techniques, including Support Vector Machine (SVM), Gaussian Process Regression (GPR), Random Forest Regression (RFR) with 100 trees, and Monte Carlo (with 30,000 iterations). Table 1 shows the necessary running time of the different surrogate models. Also, the computed mean square error (MSE) of reflection coefficient (RC) and transmission coefficient (TC) for different models are reported in this table.

**Table 1:** The necessary running time and MSE of different models for the last case.

	SVM	GPR	RFR	MC	LS-SVM
*t* (sec.)	7.7788	12.1689	20.3696	1350.2306	6.3330
MSE (RC)	0.0215	0.0148	0.0289	0.0098	0.0172
MSE (TC)	0.0199	0.0030	0.0268	0.0144	0.0034

According to the table, the LS-SVM efficiency is higher than that of the other ones. The Monte Carlo technique requires a huge amount of time. For the reflection coefficient, the mean square error of MC is better than that of other ones. However, for the transmission coefficient, the accuracy of GPR and LS-SVM is in the lower range.

## Conclusion

In this paper, the application of the LS-SVM method in crosstalk sensitivity analysis of microwave coupled-line structures is introduced. This method is very suitable both for design optimization and stochastic analysis of tolerance examinations due to the fabrication imperfection. The LS-SVM method uses a set of linear equations instead of a convex quadratic programming, which leads to significantly reduced the computational cost compared to those of the well-known Monte Carlo (MC) procedure. To show the performance of the LS-SVM, a few case-studies including uniform, and non-uniform microwave coupled-line structures are considered. For each practical case, the geometrical parameters of the coupled line are assumed to be randomly distributed using the Latin Hypercube function and the variation range of each parameter is set to ± 50% around the central value. Then, the frequency response of the coupled-line is determined for each value and an uncertain area of the response is attained. The obtained results are employed to estimate the expectation values of the associated crosstalk. Moreover, for each structure, the crosstalk Probability Density Function (PDF) is accurately anticipated and compared with those obtained by MC method. The results show that LS-SVM method predicts the target values with an acceptable accuracy and also, the obtained PDF of the crosstalk agrees well with those obtained by MC procedure with 30,000 iterations.

## Data Availability

The datasets used and/or analyzed during the current study available from the corresponding author on reasonable request.
